# Long‐range pollen transport across the North Sea: Insights from migratory hoverflies landing on a remote oil rig

**DOI:** 10.1111/1365-2656.70126

**Published:** 2025-09-17

**Authors:** Toby D. Doyle, Eva Jimenez‐Guri, Jaimie C. Barnes, Craig Hannah, Simon Murray, Christopher D. R. Wyatt, Oliver M. Poole, Karl R. Wotton

**Affiliations:** ^1^ Centre for Ecology and Conservation University of Exeter Penryn UK; ^2^ Independent Researcher UK; ^3^ Centre for Biodiversity and Environment Research, Department of Genetics, Evolution and Environment University College London London UK

**Keywords:** ecological connectivity, *Episyrphus balteatus*, insect migration, migratory hoverflies, plant–pollinator interactions, pollen metabarcoding, Syrphidae, wind trajectory analysis

## Abstract

1. Insect pollinators play a crucial role in the reproductive success of many plant species, with their movement patterns important in shaping gene flow across plant populations. Movements vary greatly from central place foragers that move over relatively short distances to migrants that move over very long distances. Of these highly mobile flower visitors, migratory hoverflies are of high ecological and economic importance as a large group of globally distributed pollinators, capable of connecting distant ecosystems through long‐range seasonal movements. However, understanding their role in pollen transport during predominantly high‐altitude migration remains challenging due to sampling constraints and the obscured identity of vectored pollen caused by post‐migratory foraging from local resources.
2. To address this, we employ ITS2 region metabarcoding to identify pollen species transported by migratory hoverflies during four distinct June or July migration events onto an oil rig devoid of vegetation and found 200 km off the coast of Scotland in the North Sea.
3. Of 121 sampled marmalade hoverflies (*Episyrphus balteatus*), 92% carried pollen, with metabarcoding of 86 individuals indicating pollen from an average of up to eight plant species per individual (range: 1–14) and 102 species in total across all four events. Dominant pollen sources included common nettle (*Urtica dioica*), black elder (*Sambucus nigra*) and meadowsweet (*Filipendula ulmaria*) alongside visits to vegetable, legume, cereal, nut and fruit species.
4. Backward wind trajectory analysis indicated northward migration in June, originating from the Netherlands, northern Germany and Denmark, over 500 km away. Conversely, migration in late July suggested southward movements from Norway, albeit with lower confidence. Forward trajectory analysis suggested potential destinations following departure from the oil rig including Norway or the Shetland Islands around 250 km away for the June migrations and Scotland for the July event.
5. Our findings highlight the capacity of migratory hoverflies to transport diverse pollen species across extensive distances, underscoring their potential role in long‐distance gene flow. Further research is essential to evaluate the ecological and agricultural implications of this phenomenon and its impact on plant communities.

## Graphical abstract

This study identifies pollen from over 100 plant species—including wildflowers, food and crops—carried by hoverflies alighting onto an oilrig 200 km off the coast during seasonal migrations. Analysis of their movements indicates a huge potential to enhance long distance gene flow between plants and increase pollination services.

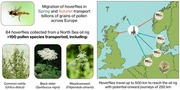

## 1 INTRODUCTION

Animal‐mediated pollination is essential for the reproduction of many flowering plants in both natural and agro‐ecosystems. Insects are the major vectors of pollen and display a great variety of forms and behaviours that influence pollen transfer and hence gene flow across landscapes (Goulson & Wright, 1997; Howlett & Gee, 2019). Understanding how this gene flow affects ecological and evolutionary processes has become increasingly important in the face of increasing habitat fragmentation, climate change and the emergence of antimicrobial and herbicide resistance. In particular, the dynamics of gene flow between populations have profound consequences for genetic diversity, adaptive evolution, range shifts and species persistence (Aguilée et al., 2016; Rojas López et al., 2022). A major element influencing gene flow is the distance over which pollinators travel, and this can have a strong effect on plant ecology and evolution.

Pollinator movements are often modelled over short distances, with long‐distance movements rare but important for inter‐population gene flow (Mola & Williams, 2019; Skogen et al., 2019). Factors such as pollinator size and hairiness may affect the quantity of pollen collected and transferred while the time between flower visits, which increases with distance travelled, may reduce pollen deposition through ingestion, grooming and loss of adhesion (Richards et al., 2005; Roquer‐Beni et al., 2022; Stavert et al., 2016). The foraging behaviour of Hymenoptera species is best studied, with the distance travelled for most social and solitary species, as central place foragers, limited by the need to return to nest sites. Honeybees generally forage the furthest at approximately 17 km from the colony (Steffan‐Dewenter & Kuhn, 2003) while the carpenter bee *Xylocopa flavorufa* has been shown to travel up to 6 km from nest sites (Pasquet et al., 2008). An exception may be seen in bumblebee queens that have been recorded actively migrating and may move over several hundred kilometres (Fijen, 2021). Non‐central place foragers are not required to return to a particular location and may forage continuously and often over long distances; for example, the fig wasp, *Ceratosolen arabicus*, may travel up to 160 km on wind currents to pollinate geographically isolated fig trees (Ahmed et al., 2009).

At the extreme end of long‐distance pollen vectoring are the migratory pollinators, which include species from Lepidoptera, Diptera and some Hymenoptera. These species migrate to take advantage of seasonal resources; boost their reproductive success; avoid habitat decline caused by temperature shifts, changes in food availability or disease risk; and to find suitable overwintering sites (Chapman et al., 2015). While these strategies have a huge advantage to insect migrants, there are also costs associated with this strategy including changes in reproductive status and death through predation or exhaustion (Herman & Tatar, 2001; Satterfield et al., 2020). Indeed, there are many strand line reports of insect migrants that succumbed during migration over water (Fisler & Marcacci, 2023; Hawkes et al., 2023). Although studying the movement of central place foragers is already a challenge, obtaining data from migratory individuals that do not return to a central nest site and move continuously over great distances has rarely been achieved. Despite this, our understanding of migratory insects' movements has been bolstered in recent years due to advances in genomic, radar, radio tracking, isotopic, wind trajectory and metabarcoding techniques that together, and particularly when combined, have significantly increased the sophistication and accuracy in determining insect migratory routes (Clem et al., 2023; Gorki et al., 2024; Hallworth et al., 2018; Reynolds et al., 2024; Suchan et al., 2018; Wotton et al., 2019). Pollen metabarcoding, utilising the ITS2 region and backward wind trajectories have proved particularly important to understand the long‐distance vectoring of pollen by migrants, with recent studies tracking painted lady butterflies (*Vanessa cardui*) across Europe and the Middle East (Gorki et al., 2024; Suchan et al., 2018) and during long‐distance dispersal events across the Atlantic Ocean (Suchan et al., 2024). Identification of pollen from these butterflies revealed a diverse set of species on their bodies, including plants with restricted ranges that could be used to assign their geographic origins (Gorki et al., 2024; Suchan et al., 2018, 2024). Despite this significant advance, the identification of plant species vectored solely during migration is obscured by local foraging on arrival and, therefore, collection of migratory individuals, that is, during migration but before arrival and foraging, is needed to unequivocally disentangle pollen loads carried during migratory movements from those collected locally.

Dipteran migrants have been understudied but contain a great diversity of species with an unsurpassed range of ecological roles. These include many pollinators, with dipterans often comprising the majority of individuals (up to 90%) in some diurnal bioflows (Hawkes et al., 2024, 2025; Hawkes, Walliker, et al., 2022; Hawkes, Weston, et al., 2022). Within the Diptera Order, the hoverflies (Syrphidae) are the most well‐studied family of migrating insects with more than 210 species thought to migrate seasonally across every continent except Antarctica (Hawkes et al., 2025). Hoverflies are widely recognised as the second most important group of pollinators globally, visiting more than 70% of wildflowers and 52% of global food crops with an estimated economic value of US$300 billion per year (Doyle et al., 2020). In addition, many are also excellent pest regulators (Tenhumberg, 1995) and decomposers (Rotheray & Gilbert, 2011). Migrant species tend to be highly abundant and generalist pollinators (Luder et al., 2018; Rodríguez‐Gasol et al., 2020; Wotton et al., 2019) and are thought to be important for the persistence of isolated plant populations (Pérez‐Bañón et al., 2007).

Movement patterns of migratory hoverflies are best understood on the western European landmass where seasonal influxes occur in spring as a multigenerational spread into northern regions from early May, followed by often huge southward migration in a single generation from late July and August to October (Aubert, 1962; Gatter & Schmid, 1990; Hawkes et al., 2024; Hawkes, Walliker, et al., 2022; Hawkes, Weston, et al., 2022; Lack & Lack, 1951; Wotton et al., 2019). Long‐distance pollen transfer by these hoverflies has been observed following a 100 km crossing of open water between the Middle East and Cyprus (Hawkes, Walliker, et al., 2022), while morphological studies of pollen have described up to three plant species carried by *E. balteatus* and *Eupeodes corollae* migrating through a mountain pass in Switzerland in the autumn (Wotton et al., 2019). Other studies using pollen morphology and DNA metabarcoding from migrating *E. balteatus* caught in a light trap on the Island of Beihuang, 40–50 km from mainland China, revealed that 32% of 1014 individuals were carrying pollen from 46 plant species, with most carrying only a single species (Jia et al., 2022). While these results from China are broadly in agreement with the few observations from Switzerland, the collection of individuals from light traps containing many other migratory species known to be pollinators, particularly moths, raises the possibility of the loss of pollen grains in the trap or cross‐contamination from other species. Despite the low diversity found on the hoverfly bodies by Jia et al. (2022), pollen metabarcoding on gut contents by the same authors found a ninefold increase in the number of species as compared to those seen on the body, indicating the visitation of a more diverse collection of flowers. Interestingly, more plant taxa were identified in the migratory populations than in field‐collected non‐migratory populations, suggesting that migratory behaviour shapes flower visitation networks, increasing the diversity of flowers visited (Jia et al., 2022).

Here, we set out to investigate the pollen species transported by migratory hoverflies during migration events to assess their potential for long‐distance pollen vectoring. We utilise metabarcoding of pollen from the bodies of migrating *E. balteatus* hoverflies caught on an oil rig located 200 km off the coast of Aberdeen and devoid of any flowering plants. This approach allows us to unequivocally identify pollen vectored during migration as the oil rig is isolated from local pollen sources.

## 2 METHODS

### 2.1 Sampling

Insects were collected either in June or July over 3 years between 2021 and 2023 from a North Sea oil rig located approximately 200 km northeast of Aberdeen, United Kingdom, latitude 58.047722, longitude 1.136556 (sample information can be found in Supporting Information File S2). These sampling dates overlap with evidence from radar data showing northward (spring) directed flight of hoverflies during early and late June, followed by southward (autumn) directed flight in late July (Wotton et al., 2019). Due to logistical and time constraints, hoverflies were sampled randomly, and samples were placed into a tube containing a new 0.5 g silica gel pack (RS components); hoverflies were coaxed/nudged into these tubes to avoid contamination. Tubes were briefly frozen at −20°C to sacrifice the insect, then stored at room temperature. Insects were identified using a ×10 hand lens without opening the lid of the tube to ensure no cross‐contamination. Four distinct movement events were sampled: (1) 24th–25th of July 2021 event—hoverflies present at 06:00 on the 24th and absent by the end of the 25th of July; (2) 30th of June 2022 event—hoverflies present at 09:00 with numbers decreasing throughout the day; (3) 10th of June 2023 event—hoverflies present at 10:00 and completely absent at 17:00; and (4) 12th of June 2023 event—hoverflies present at 06:00 and completely absent by 17:00 on the 13th of June 2023.

### 2.2 Pollen extraction

Pollen isolation and DNA amplification were performed at Penryn campus, Cornwall, United Kingdom, during winter 2024 to avoid pollen contamination. We used PCR master mix Phire Plant Direct Polymerase (Thermo Fisher Scientific) to skip out the DNA extraction process, as described in Suchan et al. (2018). Pollen isolation was performed in a clean category 2 (CAT2) hood. All consumables, equipment and samples were surface sterilised by UV and Chemgene disinfectant wipes (Star lab) prior to being introduced into the hood. Pollen isolation was performed in five batches, with an open tube containing 50 μL sterile 0.1% SDS buffer for every batch as control to determine any contamination. Flies were transferred from the sampling tube to a new tube containing 50 μL sterile 0.1% SDS buffer. After all flies were transferred, the blank tube was closed and subsequently processed as a normal sample. Pollen was washed off each individual by vortexing for 30 s. The resultant solution was removed and placed into a high‐impact screw capped tube and dried using a vacuum concentrator. The obtained pellet was diluted with 20 μL of Phire plant direct dilution buffer and homogenised with three to five zirconia beads at 5 m/s for 1 min.

### 2.3 NGS library construction

Library construction was based on the protocol used in Suchan et al. (2018). We used the ITS2 region to amplify DNA using the pollen metabarcoding primers ITS‐S2F and ITS‐4R (Chen et al., 2010; Innis et al., 1990). The first PCR amplification was prepared with primers tailed with Illumina adapters (Table S1) in a CAT2 hood. A PCR was performed for samples and the five blanks totalling 96 samples, including a PCR‐negative control. Each sample PCR was performed in triplicate to avoid reaction biases (Sickel et al., 2015; Suchan et al., 2018). Each reaction consisted of 1 μL of pollen sample, 25 μL of Phire Plant Direct Polymerase Mix (Thermo Scientific) and 0.5 μM of each primer made up to 50 μL with nuclease‐free water with the following PCR programme: 98°C for 5 min, 20 cycles of denaturation at 98°C for 40 s, annealing at 60°C for 40 s and elongation at 72°C for 40 s and final extension step at 72°C for 5 min. After the first PCR, triplicate PCR reactions for each sample were combined and mixed followed by clean‐up and size selection steps using 0.95× ratio of Nucleomag NGS clean‐up and size selection beads (Macherely‐Nagel GmbH & Co). A second PCR was performed with Illumina indexing primers to produce 96 uniquely tagged samples for a library. Indexing was performed by the University of Exeter sequencing centre. A 25‐μL reaction was made for each sample using 12.5 μL Nextera XT index kit (Illumina), 5 μL unique indexing primer (Supporting Information File S1) and 7.5 μL DNA template with the following programme: 95°C for 3 min, 12 cycles of denaturation at 95°C for 30 s, annealing at 55°C for 30 s and elongation at 72°C for 30 s, with a final elongation step at 72°C for 5 min. This second PCR was cleaned up using ×0.8 ratio of AMPure XP beads (BeckhamColuter). Libraries were quality controlled on a Tapestation D100 (Agilent) and QuantiFluor® ONE dsDNA System (Promega) and pooled and then cleaned up using 0.8× ratio of AMPure XP beads, followed by a last quality control check using the Tapestation D1000. Pooled samples were run on Illumina MiSeq using a v3 Nano 300 paired‐end flow cell (500 cycles). MiSeq raw reads were deposited on the SRA/NCBI under BioProject ID PRJNA1240305.

### 2.4 Pollen sequence data analysis

Pollen species found on each hoverfly were identified using a custom pipeline based on Suchan et al. (2018) and designed in collaboration with Eco‐Flow (https://github.com/Eco‐Flow/pollen‐metabarcoding; release v2.0.2; grant code: BB/X018768/1), using the workflow manager Nextflow (v24.10.1) (Di Tommaso et al., 2017) and following nf‐dcore structure and best practices (Ewels et al., 2020). Based on the BCdatabaser—its2.viridiplantae.EUgenera.2019‐07‐19 database from Keller et al. (2020), the pipeline classifies pollen sequences up to the species level and includes the number of reads. In brief, the pipeline takes raw fastq reads and trims the adaptors using cutadapt v4.6 (Martin, 2011); pair‐end reads were then merged using PEAR v0.9.6 (Zhang et al., 2014). Fastq files were filtered and dereplicated using VSEARCH v2.21.1 (Rognes et al., 2016) before using VSEARCH SINTAX to summarise the matches to the input database. Summary results were calculated using R, using a custom R_PROCESSING module, into output .tsv files of database matches, as well as pie charts for kingdom to species classifications.

For our analysis, raw reads were trimmed by the University of Exeter sequencing centre using cutadapt (Martin, 2011), before completing the steps outlined above. Some of the flags for each programme were changed, setting the forward and reverse primer strings (‐‐FW_primer ‘ATGCGATACTTGGTGTGAAT’ ‐‐RV_primer ‘GCATATCAATAAGCGGAGGA’), in addition to the VSEARCH v2.21.1 parameters of maximum expected error to 50% (‐‐fastq_maxee 0.5), the minimum length to 250 bp (‐‐fastq_minlen 250), the maximum Ns parameter to zero (‐‐fastq_maxns 0) and the fasta width parameter to 0 (‐‐fasta_width 0), in keeping with previously published work (Suchan et al., 2018). We also set the dereplication strand to the ‘plus’ strand and set VSEARCH sintax to ‘both’ strands setting and a cut‐off of 95% (‐‐derep_strand ‘plus’, ‐‐sintax_strand ‘both’, ‐‐sintax_cutoff 0.95). The input reads were listed in a sample sheet (‐‐input InputDoyle25), provided here (Supporting Information File S3). The input sample sheet is comma separated, listing the sample name, followed by forward and then reverse reads. We used the BCdatabaser—its2.viridiplantae.EUgenera.2019‐07‐19 plant database from Keller et al. (2020). The pipeline can be found at the GitHub repository https://github.com/Eco‐Flow/pollen‐metabarcoding and is replicable with a one‐line command, if you have docker and nextflow installed on your machine. The output file can be found in Supporting Information File S2.1.

### 2.5 Wind trajectories

Backward and forward trajectories were generated using the Hybrid Single‐Particle Lagrangian Integrated Trajectory (HYSPLIT) dispersion model (Stein et al., 2015) utilising NCEP/NCAR reanalysis data at four altitudinal layers (100, 300, 500 and 1200 m above‐ground level (m AGL)). These models simulate the dispersion and trajectory of substances transported and dispersed through the atmosphere, over local to global scales (Stein et al., 2015). Trajectory plots were generated using a pipeline developed by Suchan et al. (2024) which can be found on the GitHub repository https://github.com/etd530/Hysplit_R_interface. We ran 24‐h backward and forward trajectories for every hour over a 12‐h period based on the time of first hoverfly sighting on the oil rig. Therefore, for each movement event, 12 trajectory lines were plotted for each altitudinal layer, one every hour from the start time. Distance travelled was estimated using the wind trajectory outputs to map the likely route taken by the hoverflies. Wind data from these models were used to calculate the mean wind speed over the three altitudinal layers, which was included with a minimum and maximum hoverfly self‐powered speed of 1.2 km/h based on flight mill data (Doyle et al., 2025) and 7.2 km/h based on natural cruising speed data (Gao et al., 2020; Thyselius et al., 2023), creating a minimum and maximum hoverfly ground speed. These ground speeds were used to estimate journey duration to reach the oil rig.

### 2.6 Plant species and distributions

To determine distributions of barcoded plant species, we utilised three online databases: GBIF, Euro+Med and POWO Kew; details can be found in Supporting Information File S2.2. We examined each sample where plant identity to the species level met a probability of ≥0.95 with ≥10 reads (Gorki et al., 2024; Suchan et al., 2024). Plants that met this threshold were run through all plant distribution databases; those plant species that demonstrated a restricted distribution within Europe were then compared with the backward wind trajectories. Pollen diversity analysis of the data was carried out in R version 4.3.0 (R Core Team, 2021). Normality of the data was assessed using the Shapiro–Wilk test, followed by the construction of normal linear models and Tukey's range test. Additional analysis was performed using chi‐squared tests and one‐way ANOVA. To compare pollen species composition, we pooled samples by movement dates and sex and calculated the Wilson & Shmida Beta diversity index (Wilson & Shmida, 1984) measuring the change in diversity from one movement to another and between sexes.

## 3 RESULTS

### 3.1 Composition of sampled migrating hoverflies

**FIGURE 1:**
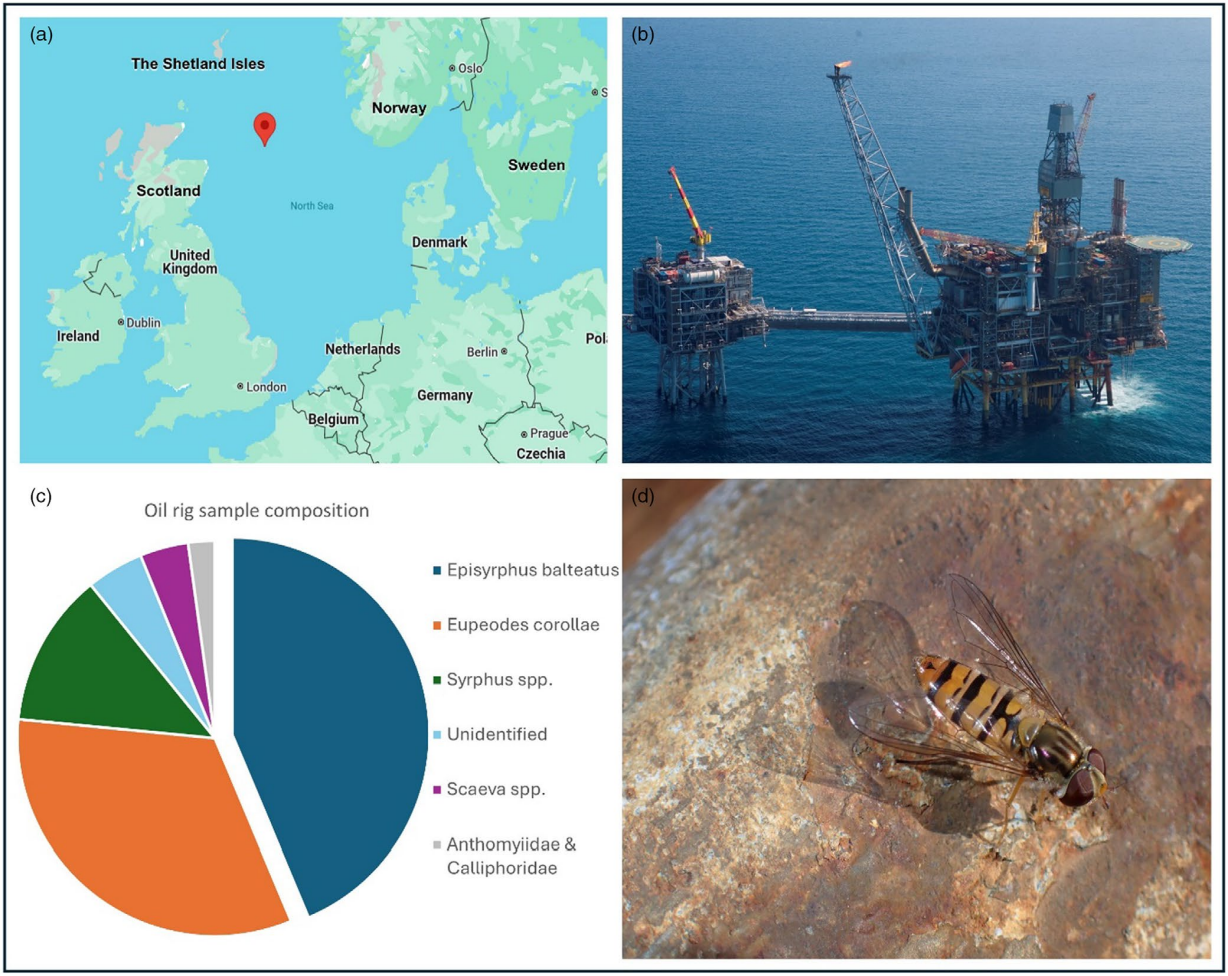
Situation of the oil rig located in the Britannia oil field and composition of migratory fly samples. (a) Location of the oil rig (Google maps), (b) aerial view of the oil rig where migratory flies were collected. (c) Species composition of migratory flies collected during June and July over 2021–2023. (d) Migratory *E. balteatus* female pictured on the oil rig (photo Craig Hannah).

Thousands of insects arrived onto the North Sea oil rig, situated 200 km off the coast of Scotland (Figure 1a,b), over a 3‐year period from 2021 to 2023, of which 277 flies were individually caught for analysis. Seven hoverfly species were identified, along with a hoverfly genus that was not identified to species level due to discolouration. In addition, flies from the families of Calliphoridae and Anthomyiidae plus some unidentified small (>2 mm) flies were found (Figure 1c; Supporting Information File S2). *E. balteatus* (Figure 1d) dominated the sampled animals with 21 males and 98 females, making up 43.7% of the individuals. *E. corollae* was the second most collected species with 15 males and 76 females, making up 32.9% of the sampled animals.

### 3.2 Pollen metabarcoding

We identified pollen grains carried on the bodies of hoverflies using DNA metabarcoding, sequencing the ITS2 region. Pollen extraction was performed only in *E. balteatus* and was detected by PCR on 92% of the individuals (111 of 121) (Supporting Information File S2). Pollen metabarcoding was conducted on 90 hoverfly samples, plus five extraction controls and one negative PCR control; see methods. Post‐analysis, only one control showed amplification with 18 reads of one plant species, *Conringia orientalis*, a species which was found in one other sample and was removed from all analyses. Eighty‐four samples were successfully run through our pipeline with the resultant plant sequences classified to the species level using the ITS2 database BCdatabaser (Keller et al., 2020). A range of 1–14 plant species was identified for each insect and a total of 102 plant species recognised overall (Table 1). Across all movement events, common nettle (*Urtica dioica*) was the most found plant species, on 50 flies, followed by black elder (*Sambucus nigra*) on 26 hoverflies and meadowsweet (*Filipendula ulmaria*) on 18 hoverflies.

**TABLE 1:** Higher represented pollen species distributions.

Migration event	Number of hoverflies	Mean species richness (SD in brackets)	Range of plant species per hoverfly	Total plant species	Top 3 most common pollen species	Number of hoverflies carrying each species
12/06/2023	14	8.1 (+ − 3)	2–14	50	*Urtica dioica*; *Sambucus nigra*; *Alopecurus myosuroides*	10; 9; 7
10/06/2023	18	6.2 (+ − 3.1)	2–12	43	*Urtica dioica*; *Sambucus nigra*; *Dactylis glomerata*	13; 13; 8
30/06/2022	16	3.6 (+ − 2.1)	1–8	30	*Urtica dioica*; *Holcus lanatus*; *Castanea sativa*	12; 4; 4
24–25/07/2021	36	3.1 (+ − 1.4)	1–6	35	*Filipendula ulmaria*; *Urtica dioica*; *Potentilla erecta*	18; 15; 8
Total	84	4.7	1–14	102	—	—

*Note*: A full list of plant species can be found in Supporting Information File S2.3.

### 3.3 Flower characterisation

**FIGURE 2:**
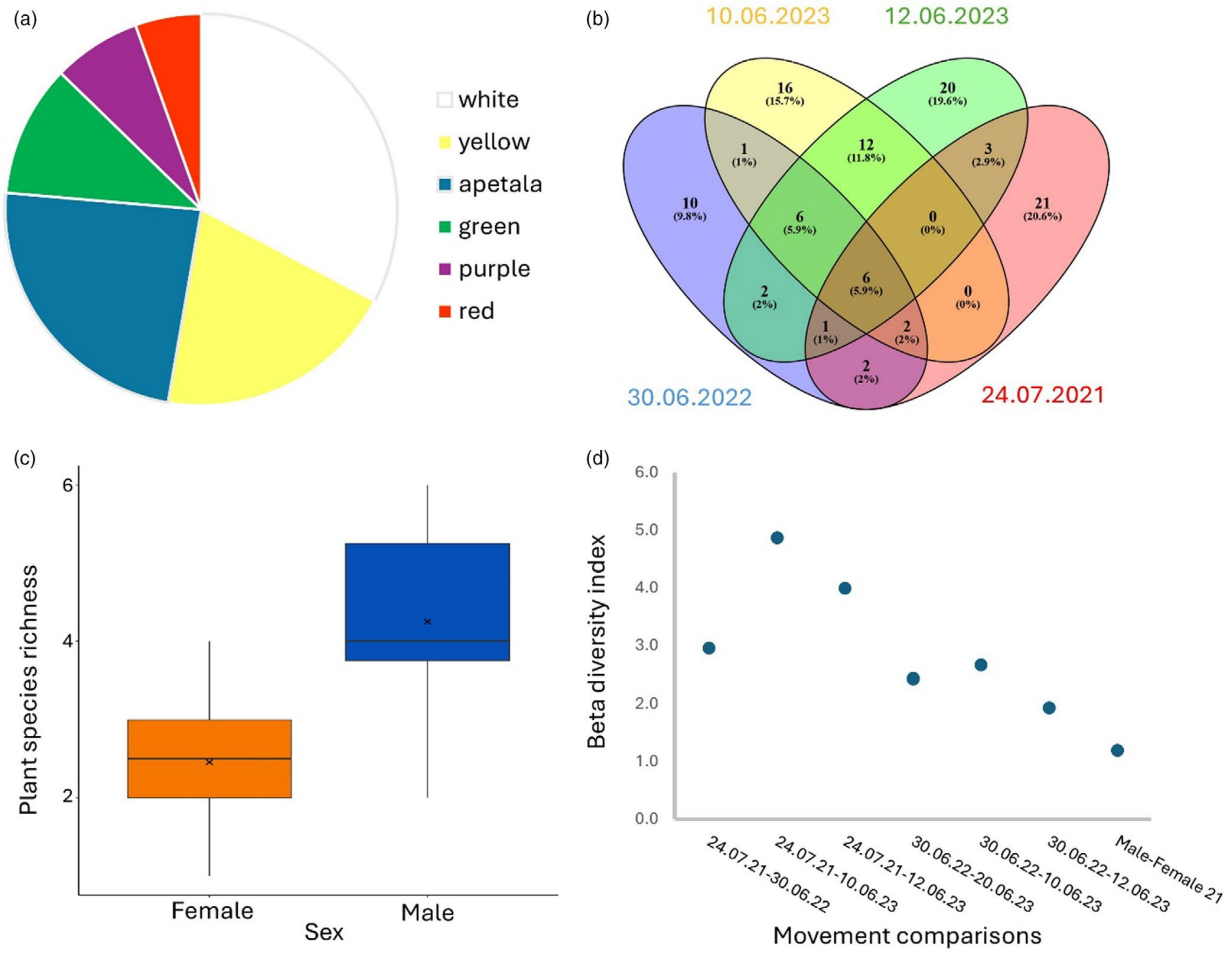
(a) Flower colour of all plants identified. (b) Venn diagram displaying the shared plant species between movement events. (c) Average plant species richness in female and male hoverflies for 2021 movement event. x denotes the mean; horizontal lines show the median and 25th and 75th percentile; vertical lines represent 0th and 100th percentile. (d) Wilson & Shmida Beta diversity index for all movement events and between males and females in 2021; a higher diversity index represents greater differences.

Of the 102 plant species that were detected, 55 species were visited by two or more flies. Nine plants were visited by more than 10 hoverflies, 21 plants by between 4 and 9 hoverflies and 12 plants visited by three hoverflies. These 55 plant species belong to 27 families, with Poaceae being the most common family (11 plants), followed by Rosaceae (5 plants) and Apiaceae (4 plants). Most of the flowers are either single (or pseudanthium) (18%) or disposed in a corymb‐like structure (29%). The most common colours were white (32%) or yellow (18%) with a considerable proportion having no petals, being either grasses or catkins (18% and 5%) (Figure 2a). Of the plants found on more than one hoverfly, only four had deep stamens, the rest having open flowers with readily accessible stamens.

### 3.4 Plant species by migration event and sex

The average number of plant species carried on the surface of each hoverfly was significantly different for each movement date (normal linear model: *F*_80,83_ = 20.35, *p* = <0.001; for detailed analysis, see Table S2 and Supporting Information File S2). On average, six and eight species were found on the June 2023 events versus three and four for the June 2022 and July 2021 events, respectively. Plant species shared between movement events ranged from 1 to 12 species with the 10th and 12th of June 2023 events sharing the most plant species (Figure 2b; Supporting Information File S2.3). In contrast, high numbers of plant species (10–21) were unique to each movement. Within all movement events, there were more female *E. balteatus* collected than males (X‐squared = 25.805, df = 1, *p*‐value = <0.001; Table S3) (females *n* = 64, males *n* = 18, unsexed *n* = 2). Males carried a significantly greater diversity of plant species than the females (27 vs. 35) during the July 2021 movement event (the only event with sufficient males to make a comparison; males *n* = 12, females *n* = 24), with averages of 4.25 for males versus 2.45 for females (one‐way ANOVA, *F*_34,35_ = 20.68, *p* = <0.05; Figure 2c; Table S3). Utilising the Wilson & Shmida Beta Diversity index across the four movement events and across sexes (Wilson & Shmida, 1984), we detected the highest indices in comparisons between the late July 2021 movement and each of the three June (2022, 2023) events, indicating a lower similarity between July and these other events (Figure 2d). In contrast, pairwise comparisons of each June event recovered lower indices, with the lowest values between the 10th and 12th June of 2023 events, indicating greater similarity in pollen compositions of these events (Supporting Information File S2.4). Comparisons of Wilson & Shmida Beta diversity between males and females from July 2021 recovered a high similarity of plant species carried by male and female hoverflies in a single movement event, although males carried more of these plant species on average.

### 3.5 Wind trajectories

**FIGURE 3:**
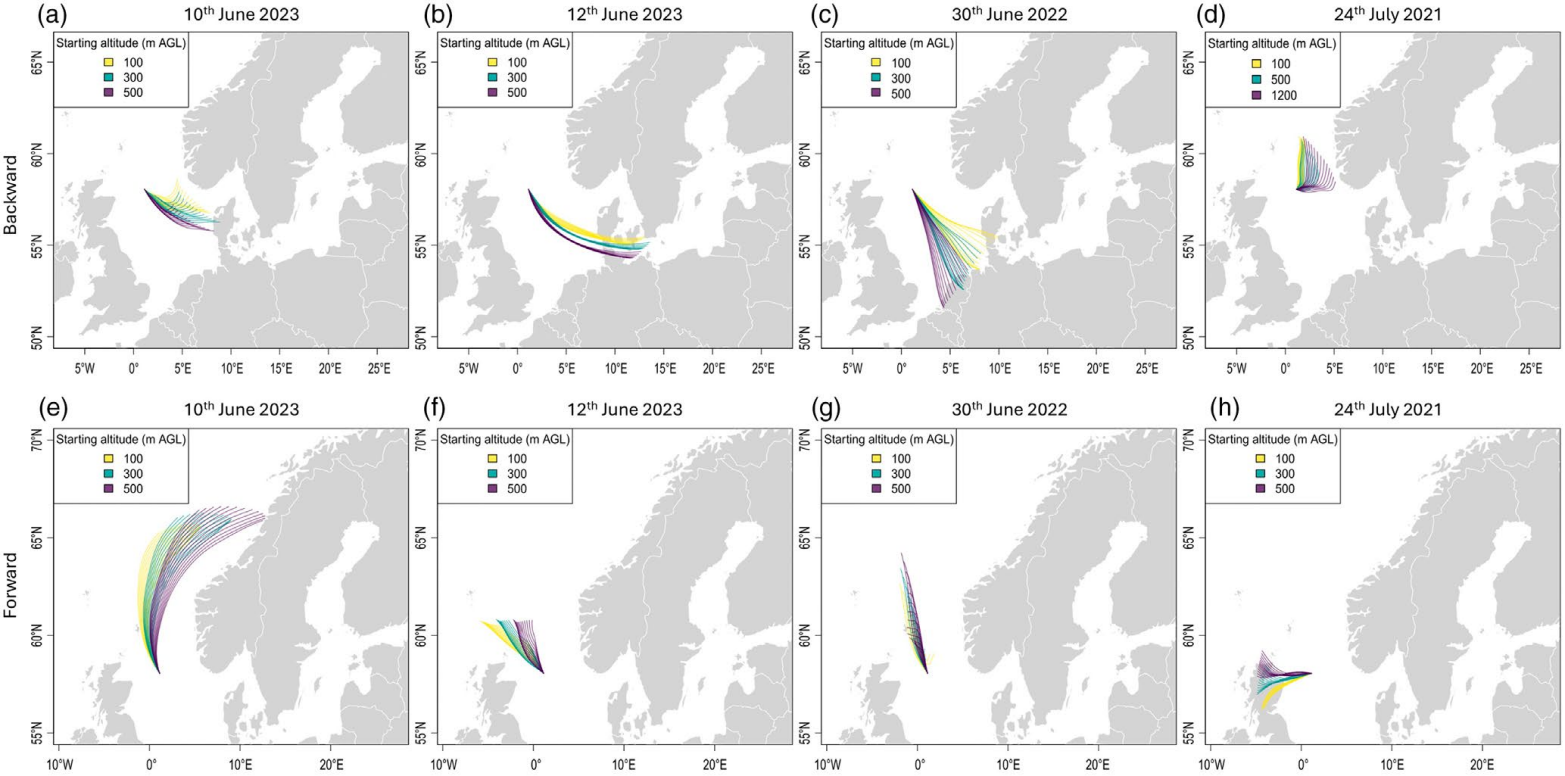
Backward (a–d) and forward (e–h) wind trajectories based on arrival times of hoverfly movement events onto the oil rig. Each coloured line shows one trajectory. Backward trajectories have a runtime of 24 h, with one trajectory calculated for every hour, 12 h before arrival time at the oil rig. Forward trajectories are the same but calculated from the time the flies left the rig. Colours show altitude (yellow, 100; green, 300; purple, 500 m AGL), apart from (d) where green represents 500 and purple 1200 m AGL.

To investigate the geographic origin of these hoverflies, we employed backward wind trajectories at four altitudinal layers: 100, 300, 500 and 1200 m AGL. Backward trajectories at lower altitudes (≤500 m AGL) on 24 July 2021 did not result wind trajectories that reached landfall; therefore, we extended the altitudinal layer to 1200 m AGL, demonstrating a ‘close to land’ trajectory. Twelve 24‐h backward trajectories were plotted, starting at the time of the first hoverflies observed on the oil rig and the 11 h before that to accommodate for the hours when no observers were present (see Table 2; Figure 3). Back trajectories run for the 10th and 12th June of 2023 and 30th June 2022 and suggest migrants originated from southeast of the oil rig, from Denmark, northern Germany and the Netherlands, with an approximate distance travelled of between 265 and 500 km to reach the oil rig (Figure 3a–c). Following arrival onto the oil rig, migrant hoverflies departed the same or next day. Forward trajectories from these June dates suggest that movement would have continued north after the hoverflies left the oil rig with potential landfalls in the Shetland islands (all dates) or Norway (10th of June) (Figure 3e–g). Back trajectories for the 24th July 2021 suggest Norway as the likely origin of these migrants, though these trajectories fail to make landfall (Figure 3d). Forward trajectories from this date suggest a subsequent landfall onto mainland Scotland 200 km to the west (Figure 3h).

**TABLE 2:** Hoverfly movement metrics.

Movement date	Time of first observation	Max distance to oil rig (km)	Average wind speed (km/h) (SD in brackets)	Hoverfly average ground speed (km/h) (range in brackets)	Estimated time interval to reach oil rig (h)	Total distance (km)
10 June 2023	1030	265	15.5 (+ − 1.4)	22.7 (17.3–22.7)	11.7–15.3	515
12 June 2023	0600	400	34.3 (+ − 1)	41.5 (36.1–41.5)	9.6–11	650
30 June 2022	0900	500	27.1 (+ − 1.1)	34.3 (28.9–34.3)	14.6–17.3	750
24 July 2021	0600	267	11.8 (+ − 1.2)	19 (13.6–19)	14–19.6	447

*Note*: Hoverfly movement dates and times of first observation showing maximum distance to oil rig based on backward trajectories. Average wind speed was calculated over all altitudinal layers and used to calculate hoverfly ground speeds assuming an additive downwind self‐powered flight speed of 7.2 km/h, ranging from 1.2 km/h (Doyle et al., 2025) to 7.2 km/h (Gao et al., 2020; Thyselius et al., 2023). The estimated time interval to reach the oil rig is the maximum distance divided by the minimum and maximum hoverfly ground speeds. Total distance is the distance travelled to reach the oil rig plus the additional distance travelled to reach land by hoverfly, based on backward and forward wind trajectories.

Based on these trajectories and the location of the closest land mass, we estimated the distance travelled for each movement event onto the oil rig to range from 265 to 500 km. Average wind speeds during these events ranged from 11.2 to 34.3 km/h allowing us to estimate ground speeds of 19 to 41.5 km/h during downwind flights given a self‐powered speed of 7.2 km/h (2 m/s) for migrant hoverflies (Table 2; Gao et al., 2020; Thyselius et al., 2023). At these ground speeds, the duration of wind‐assisted flight needed for each movement to reach the oil rig ranged from 9.6 to 19.6 h.

### 3.6 Restricted plant distribution

In total, three of 84 hoverflies carried pollen from two plant species with restricted distributions in Europe: *Festuca prolifera* and *Rumex lapponicus* (for distributions, see Figures S1 and S2). All three flies carried *F. prolifera* pollen, one individual from 10 June 2023 and two from 12 June 2023, while the same hoverfly from 10 June 2023 also carried *R. lapponicus* pollen. Within Europe, *F. prolifera* and *R. lapponicus* have a restricted distribution to Norway, Sweden and Finland (Jonsell, 2000; Tutin et al., 1964; Supporting Information File S2.2). Backward trajectory wind analysis from both the 10th and 12th of June 2023 for 12 h converges on Denmark (Figure 3a,b) while extending back trajectories to 36 h shows convergence over Norway and southern Sweden, respectively (see Figure S3). A combined information file containing sample name, movement dates, identified pollen species and trajectories can be found in Supporting Information File S2.5.

Plant species transported showing pan‐European distributions were further analysed to determine whether they were present in likely hoverfly destinations, as predicted by wind trajectory models, that is, Scotland and the nearby islands, including the Shetland and Orkney Islands. These distributions demonstrate that 70% of plant species identified on the bodies of these hoverflies are present in these areas, with the remaining 30% absent. These absent species comprise plants that are not suited to the Scottish mainland and islands or have restricted distributions. A full list of present and absent species can be found in Supporting Information File S2.6.

## 4 DISCUSSION

Hoverflies are a diverse and globally important family with a high potential for mediating long‐distance pollen transfer due to the migratory life histories of many species and their global distribution (Doyle et al., 2020; Wotton et al., 2019). Using pollen metabarcoding and wind trajectory modelling, we suggest movement of the palaearctic hoverfly *E. balteatus* over 265–500 km of open water in a single journey. Most of these individuals (92%) vectored pollen from between three and eight species on average, and from over 100 plants in total. Importantly, collection of these samples from an oil rig excludes the possibility of contamination by local flora and unequivocally identifies pollen vectored over long distances.

### 4.1 Seasonal movement patterns, geographic origins and destinations

The movement of insects across large expanses of land and water has been known for many years based on field observations (Sutton, 1969; Svensson & Janzon, 1984; Walker, 1864). Indeed, many incidental sightings of insect migration have occurred on a variety of ships and semi‐permanent oceanic structures such as shipping vessels or oil rigs (Fox, 1973, 1978; Holzapfel & Harrell, 1968). The sudden occurrence of these insect migrants is likely due to a need to rest after sustained flight and/or an attraction to far away lights in an otherwise dark landscape (Alves et al., 2019). Using wind trajectory models to predict the direction and duration of hoverflies travelling to and from the oil rig, we recover movements broadly in line with radar data described by Wotton et al. (2019). These radar data showed northward‐directed flight of hoverflies during early and late June, followed by southward‐directed flight from late July onwards (Wotton et al., 2019). Our backward trajectory models show northward‐directed spring (early and late‐June) migration in three of the movement events (Figure 3a–c). These trajectories suggest a likely origin in Denmark, northern Germany and the Netherlands, respectively. While these trajectories do not show the point where the hoverflies departed, hoverflies migrating during spring have been shown to travel with the wind to increase their ground speed (Gao et al., 2020). Conversely, backward trajectories from late July 2021 (Figure 3d) showed southward orientation, likely originating from Norway. Despite this, the only pollen identified with restricted distribution was found on hoverflies moving north from mainland Europe in both 2023 movement events (Figure 3a,b). These plants showed a restricted distribution in Norway, Sweden and Finland. However, backward wind trajectories extended to 36 h indicate Sweden as the likely origin of these hoverflies, suggesting pollen may remain on the bodies of migrants for a considerable length of time (Figure S3). However, it is important to note that these wind trajectories (Figure S3) also pass over Denmark, so it may be the case that some hoverflies from both 2023 movement events could have originated in Denmark; with the current pollen data, these possibilities cannot be distinguished.

Based on estimated origins of each hoverfly movement, we approximate the distance travelled to be between 265 and 500 km from the last point of land to the oil rig, with a flight time of between 9.6 and 19.6 h. It is unclear if hoverflies landing on the oil rig were subsequently able to reach land; however, their rapid disappearance suggests their migratory journey was continued. Forward wind trajectories show that hoverflies have the potential to make land fall after leaving the oil rig, with a likely destination of the Shetland Islands (Figure 3e–g) and mainland Scotland (Figure 3h). Interestingly, the migration of hoverflies onto high latitude islands has previously been documented. In September 2000, a variety of migratory species including five hoverfly species arrived at two geographic areas in the Faroe Islands (Jensen, 2001). Utilising meteorological data, Jensen postulated a migratory route similar to the route proposed here, with insects travelling across the North Sea on a band of high pressure. Based on forward trajectories, we can estimate that the total distance travelled by our hoverflies would be between 447 and 750 km. While long distance vectoring of pollen has been evidenced in non‐migratory fig wasps transporting pollen up to 160 km (Ahmed et al., 2009), migratory insects have a greater propensity to vector pollen over large distances. Examples include the 4200 km journey of *V. cardui* across the Atlantic Ocean from West Africa to French Guiana (Suchan et al., 2024) and hoverflies crossing of more than 100 km of sea to Cyprus, 40–60 km over the Bohai Strait in Shandong (China) (Feng et al., 2006; Hawkes, Walliker, et al., 2022; Jia et al., 2022). We believe the distances outlined here represent the longest recorded example of hoverfly‐mediated pollen vectoring.

### 4.2 Long‐distance pollen transfer

The influx of migrants carrying pollen has the potential to influence plant population dynamics. For example, the white‐lined sphinx hawk moth (*Hyles lineata*) has been shown to contribute to high gene flow and low population differentiation in many plant species (Skogen et al., 2019), whereas geographically isolated islands east of the Spanish mainland benefit greatly from the seasonal immigration of the hoverfly *E. tenax* that pollinates many native plants (Pérez‐Bañón et al., 2007). Previous studies investigating pollen vectoring in migrant hoverflies have described up to three plant species carried by migrating *E. balteatus* and *E. corollae* in the col de Bretolet, Swiss Alps, based on pollen morphology (Wotton et al., 2019), whereas migrating *E. balteatus* caught on Beihuang Island in Shandong, China, were observed carrying only one plant species, with only a few hoverflies carrying pollen from different plant species based on morphology and barcoding (Jia et al., 2022). In contrast to our expectations based on these observations, we observe that the majority (92%) of 121 migrating *E. balteatus* individuals caught on the oil rig carried pollen. These pollen grains came from a large variety of plant species: A total of 102 plants carried by 84 hoverflies, carrying between 1 and 14 plant species per individual (mean 4.7). In comparison, pollen metabarcoding of 180 larger *Eristalis* hoverflies foraging in grasslands in West Wales, United Kingdom, were found to transport pollen from 65 plant species (Lucas, Bodger, Brosi, Ford, Forman, Greig, Hegarty, Jones, et al., 2018). While these studies represent northern European habitats, studies taking place in hotspots of plant diversity such as the Mediterranean have recovered higher plant diversity on larger migrating insects, with 157 plant species found on 47 *V. cardui* migrating between Africa and Spain (Suchan et al., 2018).

With such high pollen loads, these hoverflies have the potential to provide long‐range pollination services. Remarkably, 70% of plant species found on the surface of hoverflies are also present in the areas that the hoverflies are predicted to reach based on our forward trajectories (Figure 3e–h), demonstrating a high degree of connectance with the potential to increase plant genetic diversity and transfer ecologically and agriculturally important alleles. However, to be an effective long‐distance pollinator, the pollen needs to remain viable and be deposited onto conspecific flowers. The viability of pollen varies from species to species; for example, oilseed rape (*Brassica napus* L.) and cotton (*Gossypium hirsutum* L.) are viable for up to 2 days on the proboscis of *Helicoverpa armigera* (Richards et al., 2005) whereas the viability of oilseed rape may be up to 15 days under specific temperatures and relative humidity (Jacqueline & Renard, 2002) and white oak (*Quercus alba*) keeps a 16% viability after 30 days (Codina et al., 2011). Influencing this viability are a variety of environmental conditions such as wind speed, influencing pollen detachment, precipitation, washing pollen off the surface of flies and ultraviolet light, causing irreparable damage to pollen cells (Torabinejad et al., 1998). We estimate that the hoverflies take between 9.6 and 19.6 h (Table 2) to make their crossing to the oil rig; their onward journey remains a mystery. However, considering the short time it has taken them to reach the oil rig, we believe they would carry viable pollen, though the deposition of this pollen on conspecifics remains to be investigated.

### 4.3 Plant species vectored by hoverflies

Wildflowers and crops are pollinated by a variety of wild and commercial pollinators. Hoverflies are the most dominant non‐bee pollinator known to visit 52% of world crop plants, with an estimated worth of US$300 billion per year, and 70% of animal‐pollinated wild flowers (Doyle et al., 2020). Pollination by hoverflies differs from that of bees, whereas bee foraging range is limited by hive or nest location, hoverflies are not restricted to a home range and are able to transport pollen over larger distances. We identified pollen from a number of notable food and crop species including cereals like durum wheat (*Triticum turgidum*) and various forage grasses, vegetables including cabbage (*Brassica oleracea*), onion (*Allium cepa*), potato (*Solanum tuberosum*), and Ethiopian mustard (*Brassica carinata*), the legume broad bean (*Vicia faba*), herbs such as chamomile (*Matricaria chamomilla*), nut species like sweet chestnut (*Castanea sativa*) and a number of wild fruits including wild cherry (*Prunus serotina*), dew berry (*Rubus caesius*) and black elder (*Sambucus nigra*), along with cover and green manure crops such as fiddleneck (*Phacelia tanacetifolia*). Except for black elder, these were recovered from single or low numbers of hoverflies (range 1–6). However, given the estimated billions of *E. balteatus* travelling from mainland Europe to Great Britain and back every year, this represents huge potential for the substantial vectoring of food and crop pollen (Wotton et al., 2019). Further investigation over larger areas, sampling more individuals and over a larger range of blooming periods will be important to identify their full contribution to vectoring pollen from other commercial crop and food species.

We identify a wide range of pollen from different wild plant species carried by *E. balteatus* including from forbs, trees, shrubs, grasses and sedges. Flower morphologies dictate pollen vectoring by restricting the range of pollinators that may visit a flower (Fenster et al., 2004). In our data, we see most flowers visited had inflorescences with a flattened structure (corymb‐like) or single flowers or pseudoflowers, which promote visits by hoverflies (Branquart & Hemptinne, 2000; Lucas, Bodger, Brosi, Ford, Forman, Greig, Hegarty, Neyland, et al., 2018). We also see a strong bias towards white flowers, and yellow flowers being the second most favoured colour (Figure 2a). This contrasts with previous studies which found that yellow was the preferred flower for *E. balteatus* (Kugler, 1950; Sutherland et al., 1999). The most transported pollen species in terms of number of hoverflies and movement events belongs to the common nettle. Despite this being a wind‐pollinated plant, it is occasionally insect‐pollinated (Taylor, 2009), and indeed, *Eristalis* species have been found to transport its pollen (Lucas, Bodger, Brosi, Ford, Forman, Greig, Hegarty, Neyland, et al., 2018). Interestingly, *E. balteatus* larvae also feed on aphids infesting common nettle plants (Alhmedi et al., 2009), which may explain adult visitation. In addition, one of the most common families identified was of the grasses Poaceae and together with pollen from sedges and nettles suggest that wind‐dispersed (anemophilous) pollen may form an important part of the diet of migrants, as has been observed in other Syrphids (Holloway, 1976; Ssymank & Gilbert, 2008). Black elder pollen was also a predominant species transported by hoverflies and has open easily accessible flowers that produce a strong odour to attract a variety of insect pollinators (Atkinson & Atkinson, 2002). Interestingly, its berries also provide essential forage for frugivorous passerine migrant birds during autumn migration (Ottich & Dierschke, 2003). Together, these observations suggest Black elder is an important food source for migrants that could be encouraged along flyways to support migratory journeys.

### 4.4 Plant species vectored over movement events

North and southward migration occurs over several months within the northern European migratory timeline. Over a 3‐year period, we see a wide range of pollen diversity both between individuals from the same movement event and between movement events. In our most diverse movement event (12th of June 2023), we see pollen diversity ranging from 2 to 14 plant species per hoverfly with a total of 50 plant species carried by all hoverflies. Beta diversity measurements (Figure 2d; Supporting Information File S2.3) identified low similarities between early June and late July movement dates. These differences in pollen diversity likely reflect both intra‐specific variation in the flowering periods and differences in the abundances of plant species in the different geographic origins (May et al., 2017). The most similar movement events were between both early June 2023 dates (Figure 2b), where we find an increased number of shared plant species likely driven by similar plant phenologies given these events were 2 days apart. We also see a greater diversity of plant species in the 2023 movement events but a reduction in species shared across the 2021 and 2022 dates (Table 1). Potentially, this is because of spatial‐temporal changes in plant phenology (Rzanny et al., 2024), resulting in an increase in forage for those hoverflies in 2023. Measures of beta diversity between males and females identified a similar pollen composition (Figure 2d), but with males carrying significantly more pollen species on average (Figure 2c). These results mirror that of Svensson and Janzon (1984) who found that male migratory *E. corollae* carried significantly more pollen grains than female migrants with interesting differences in pollen composition. It is unclear why this should be the case, but it may reflect fundamental differences between the sexes, such as flight performance (Doyle et al., 2025), or territorial behaviour in males that may increase flower visitations (Wellington & Fitzpatrick, 1981).

## 5 CONCLUSIONS

We evidence the long‐distance transfer of pollen by migratory hoverflies across the North Sea to an isolated oil rig platform approximately 200 km northeast of Aberdeen. Utilising wind trajectory analysis, we estimate distances travelled of between 265 and 500 km from the last land mass to the oil rig. Considering the rapid departure of hoverflies from the oil rig, it is likely that these hoverflies continued their journey, resulting in an estimated total travel distance of between 447 and 750 km to reach land. Migratory hoverflies make this journey during both spring (northward) and autumn (southward) migrations. Pollen metabarcoding revealed between 1 and 14 plant species are vectored by migrating *E. balteatus*, with a diverse range of 102 plant species detected overall. The transportation of these plant species by hoverflies has the potential to enhance long distance gene flow between plants and increase pollination services. Future studies should investigate pollen viability, in addition to visitation and pollination networks following migratory events over the whole migratory period, in order to detail the ecological outcomes of long‐distance vectored pollen on destination ecosystems.

## AUTHOR CONTRIBUTIONS

Toby D. Doyle, Eva Jimenez‐Guri and Karl R. Wotton conceptualized the project and wrote the manuscript. Toby D. Doyle and Eva Jimenez‐Guri designed and performed research. Craig Hannah collected the samples. Simon Murray and Christopher D. R. Wyatt wrote the pipeline. Toby D. Doyle, Eva Jimenez‐Guri, Karl R. Wotton, Jaimie C. Barnes, Oliver M. Poole and Christopher D. R. Wyatt analysed the data. Toby D. Doyle, Eva Jimenez‐Guri, Karl R. Wotton, Jaimie C. Barnes, Oliver M. Poole and Christopher D. R. Wyatt reviewed and edited the manuscript.

## FUNDING INFORMATION

This study was supported through grants to K.R.W. from the Royal Society University Research Fellowship scheme (grant no. UF150126). T.D.D., E.J.G. and O.M.P. were supported by awards to K.R.W. from the Royal Society: a fellow enhancement award (grant no. RGF\EA\180083). J.C.B. was supported through a Biotechnology and Biological Sciences Research Council (BBSRC). S.M. and C.D.R.W. were supported through a BBSRC grant (grant no. BB/X018768/1).

## CONFLICT OF INTEREST STATEMENT

The authors declare that there is no conflict of interest.

## Supporting information


**Table S1.** ITS2‐S2F & ITS‐4R primers with Illumina adapters in bold.
**Table S2.** Summary statistics of Tukey's HSD post doc test comparing plant species carried by hoverflies for each movement event.
**Table S3.** Pollen metabarcoding summary statistics based on sex from 2021 migration.
**Figure S1.** Euro+Med (left) and GBIF (right) database distributions for *Festuca prolifera*.
**Figure S2.** Euro+Med (left) and GBIF (right) database distributions for *Rumex lapponicus*.
**Figure S3.** Backward wind trajectory‐based arrival times of hoverfly movement events onto the oil rig for the (a) 10th of June 2023 and (b) 12th of June 2023 movement events. Runtime of 36 h, with one trajectory calculated for every hour, 12 h before arrival time at the oil rig. Colours show altitude (yellow, 100; green, 300; purple, 500 m AGL).

**Supporting Information S1.** Illumina unique indexing primers for second stage PCR.

**Supplementary information S2.** *Episyrphus balteatus* samples collected.
**Supplementary information S2.1.** Pollen reads per plant species found in each hoverfly sample, using a 95% probability of plant sequence species match (Table A). Table B show these data with a filter applied for plants with 10 or more reads. All assignments that didn't reach species level were removed. These data were used for all analysis.
**Supplementary information S2.2.** Hoverfly samples with pollen from a plant with restricted geographical distribution.
**Supplementary information S2.3.** Plant species found in each date with fly sample numbers. Cells highlighted in blue show plant species shared between movement events.
**Supplementary information S2.4.** Beta diversity index between movement dates.
**Supplementary information S2.5.** Combined hoverfly information, plant species transported, movement date and estimated origin and destination locations.
**Supplementary information S2.6.** Plant species present and absent from likely destination of migratory hoverflies—Scotland, Orkney or Shetland.

**Supporting Information S3.** Pipeline sample input sheet.

## Data Availability

MiSeq raw reads were deposited on the SRA/NCBI under BioProject ID PRJNA1240305.
