# Supplementary material for: Long‐range pollen transport across the North Sea: Insights from migratory hoverflies landing on a remote oil rig
Source: J Anim Ecol. 2025 Sep 17;94(11):2267–81. doi: 10.1111/1365-2656.70126 (PMC12586786; doi:10.1111/1365-2656.70126)
Supplement: Supplementary file 1 — Table S1. ITS2‐S2F & ITS‐4R primers with Illumina adapters in bold. Table S2. Summary statistics of Tukey's HSD post doc test comparing plant species carried by hoverflies for each movement event. Table S3. Pollen metabarcoding summary statistics based on sex from 2021 migration. Figure S1. Euro+Med (left) and GBIF (right) database distributions for Festuca prolifera. Figure S2. Euro+Med (left) and GBIF (right) database distributions for Rumex lapponicus. Figure S3. Backward wind trajectory‐based arrival times of hoverfly movement events onto the oil rig for the (a) 10th of June 2023 and (b) 12th of June 2023 movement events. Runtime of 36 h, with one trajectory calculated for every hour, 12 h before arrival time at the oil rig. Colours show altitude (yellow, 100; green, 300; purple, 500 m AGL). [file JANE-94-2267-s003.docx]

Table S1. ITS2-S2F & ITS-4R primers with Illumina adapters in bold.

| ITS2-S2F | TCGTCGGCAGCGTCAGATGTGTATAAGAGACAGATGCGATACTTGGTGTGAAT |
| --- | --- |
| ITS2‐4R | GTCTCGTGGGCTCGGAGATGTGTATAAGAGACAG**TCCTCCGCTTATTGATATGC** |

Table S2. Summary statistics of TukeysHSD post-doc test comparing plant species carried by hoverflies for each movement event.

| Movement date comparison | Difference mean | Lower confidence interval | Upper confidence interval | Adjusted P value |
| --- | --- | --- | --- | --- |
| 12/06/2023-10/06/2023 | 1.976 | -0.166 | 4.118 | 0.081 |
| 24-25/07/2021-10/06/2023 | -3.111 | -4.846 | -1.376 | 0.000 |
| 30/06/2022-10/06/2023 | -2.542 | -4.607 | -0.476 | 0.010 |
| 24-25/07/2021-12/06/2023 | -5.087 | -6.981 | -3.194 | 0.000 |
| 30/06/2022-12/06/2023 | -4.518 | -6.718 | -2.318 | 0.000 |
| 30/06/2022-24-25/07/2021 | 0.569 | -1.237 | -1.237 | 0.841 |

Table S3. Pollen metabarcoding summary statistics based on sex from 2021 migration

| 2021 Migration event | Number of flies | Mean plant species richness | Range of plant species per fly | Total plant species |
| --- | --- | --- | --- | --- |
| Males | 12 | 4.25 | 2-6 | 27 |
| Females | 24 | 2.46 | 1-4 | 35 |


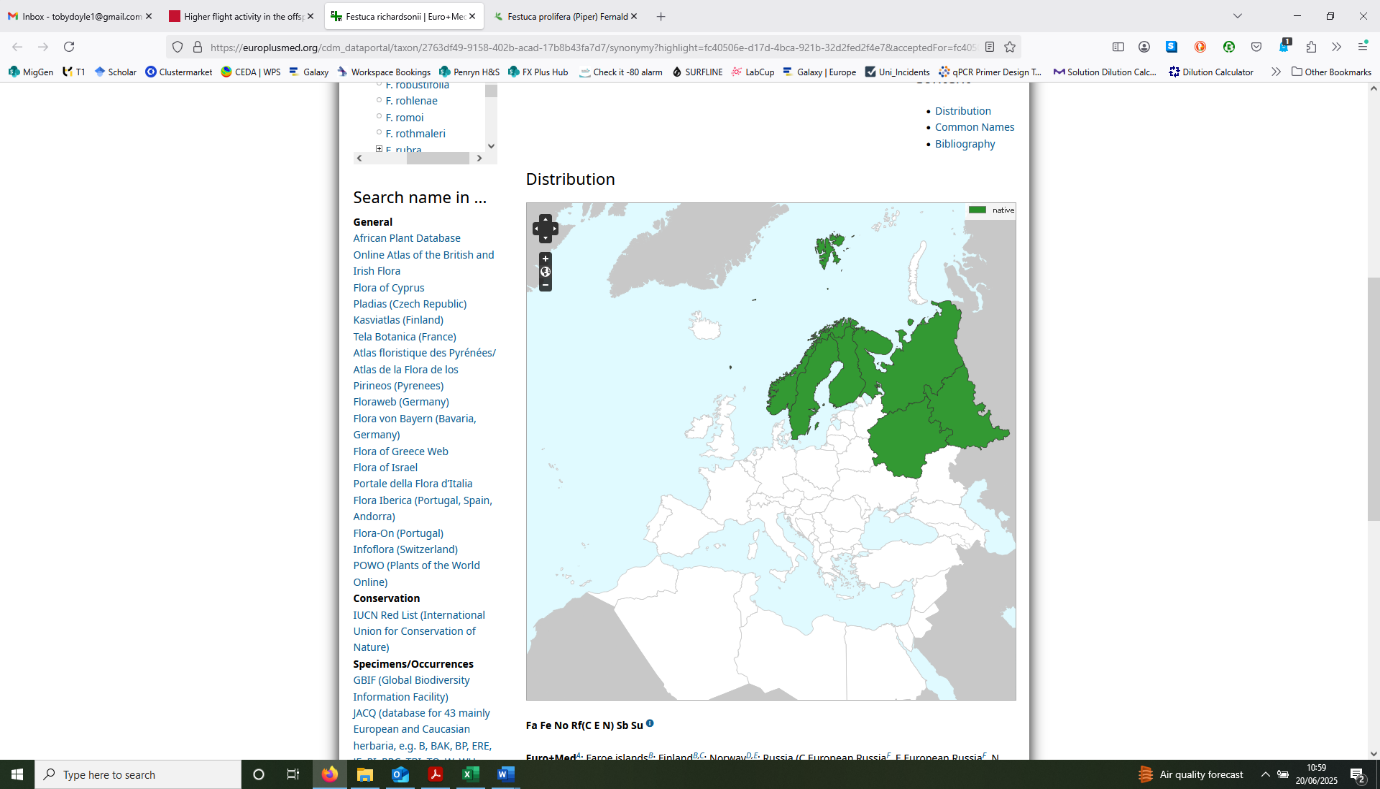

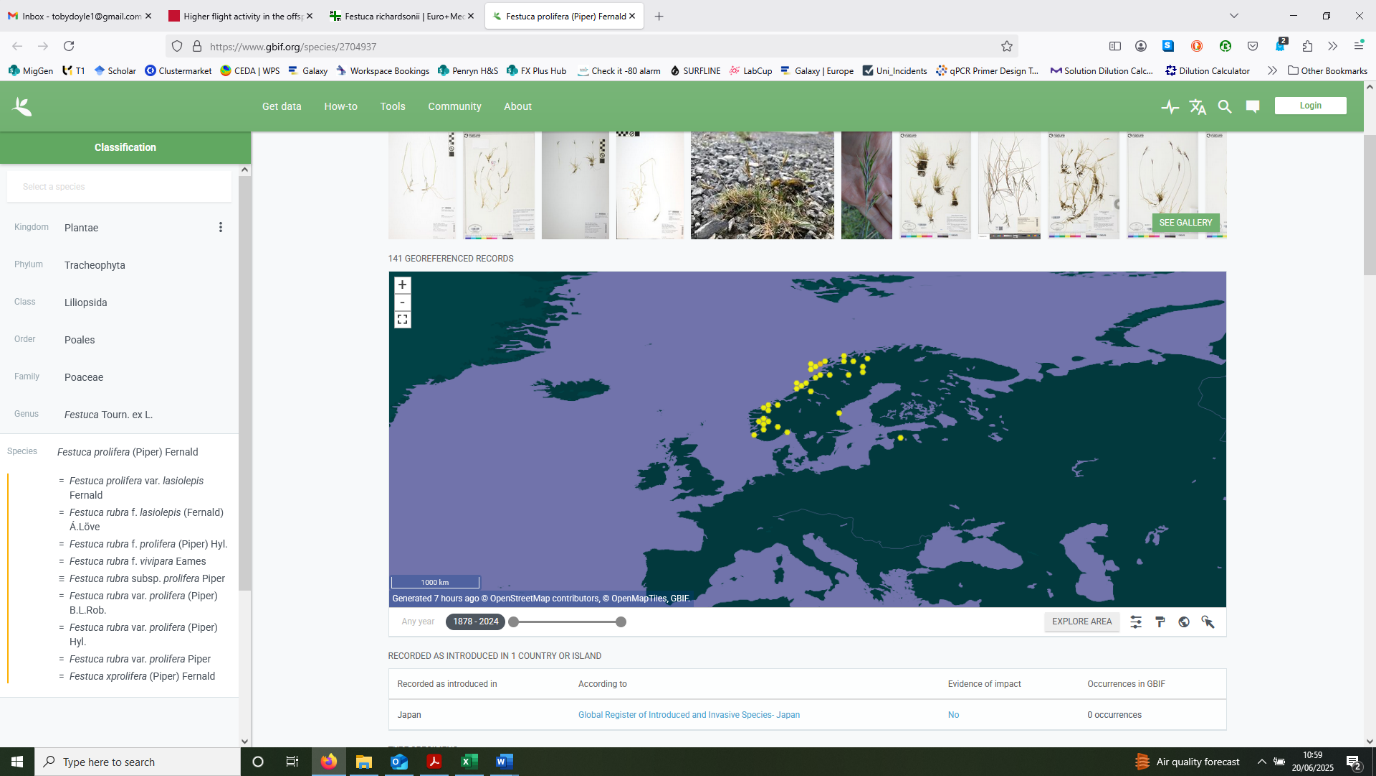


Figure S1. Euro+Med (left) and GBIF (right) database distribtuions for *Festuca prolifera*


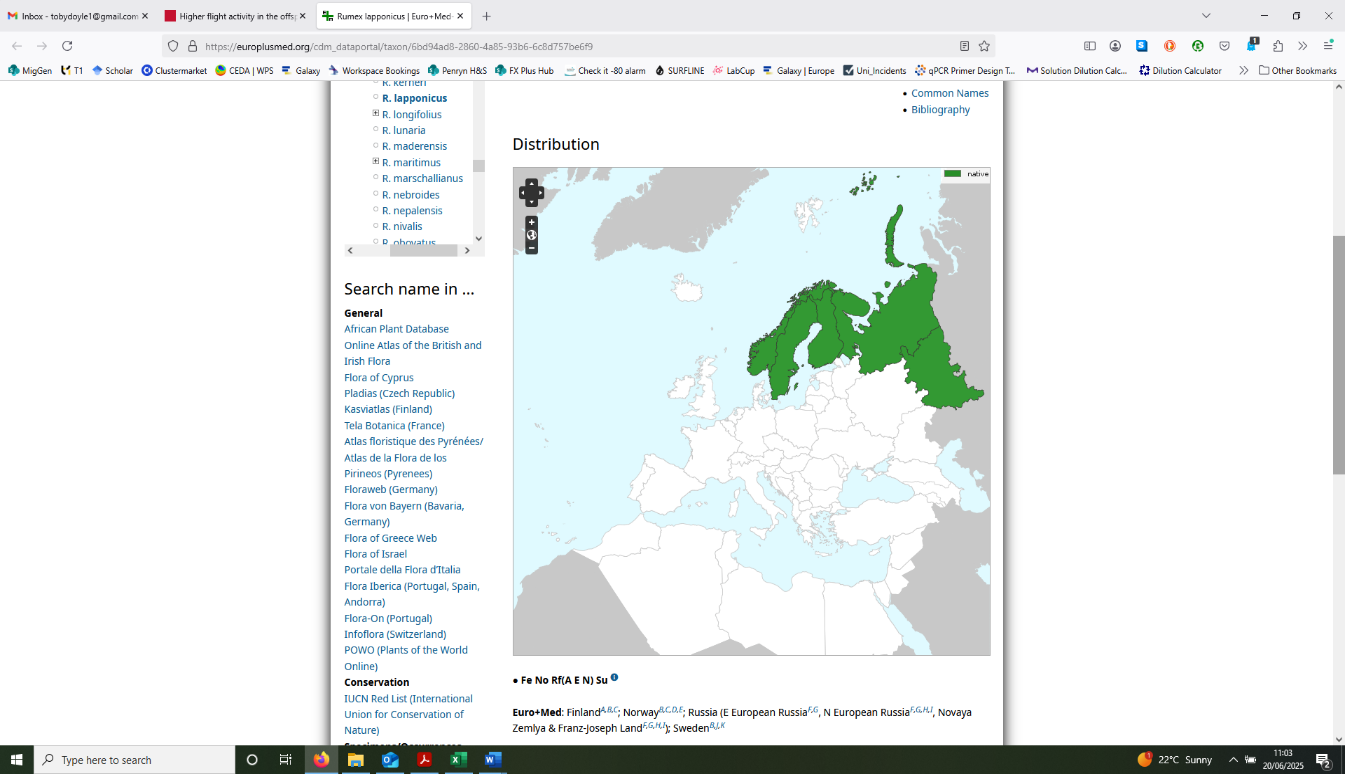

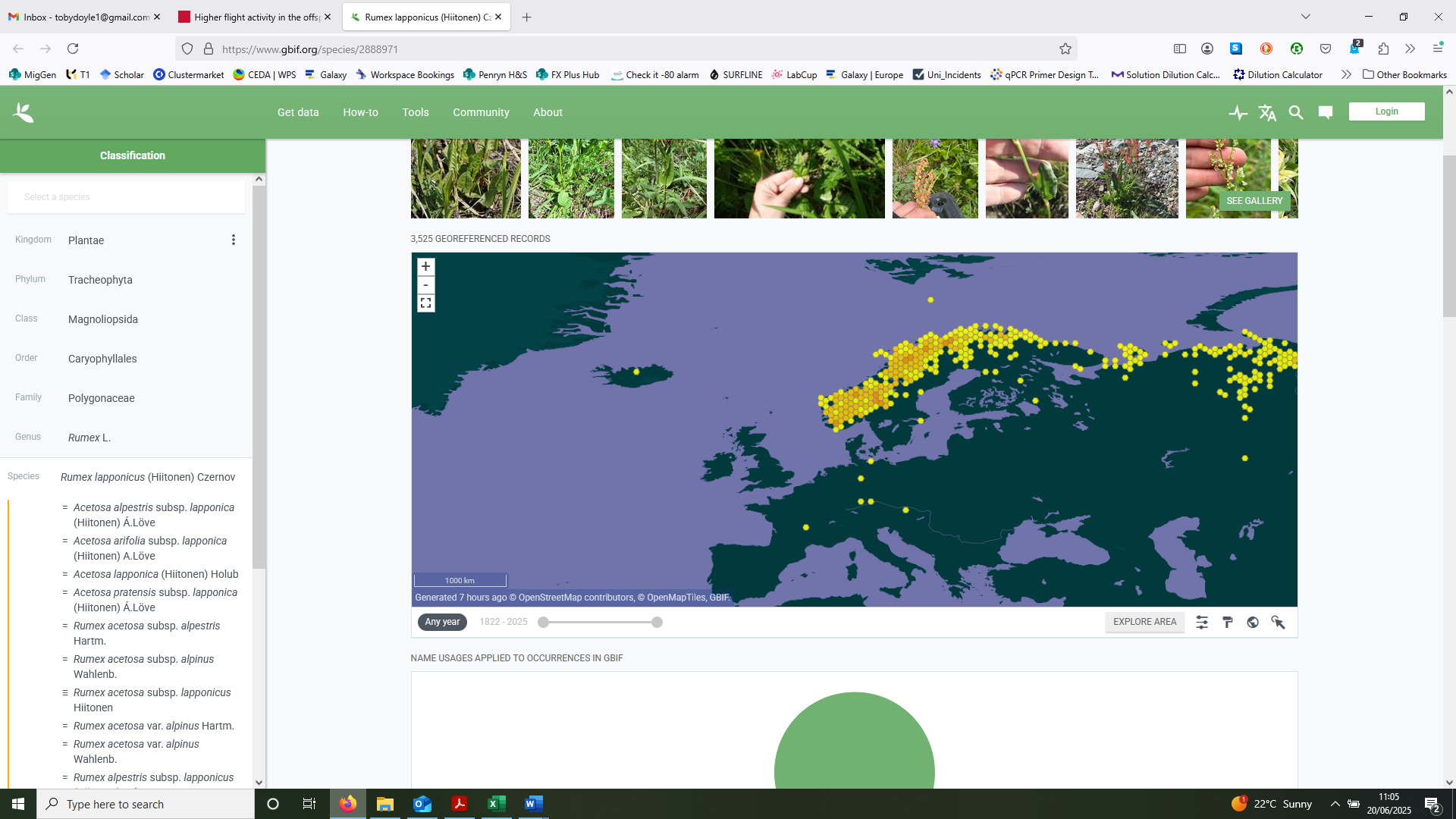


Figure S2. Euro+Med (left) and GBIF (right) database distribtuions for *Rumex lapponicus*


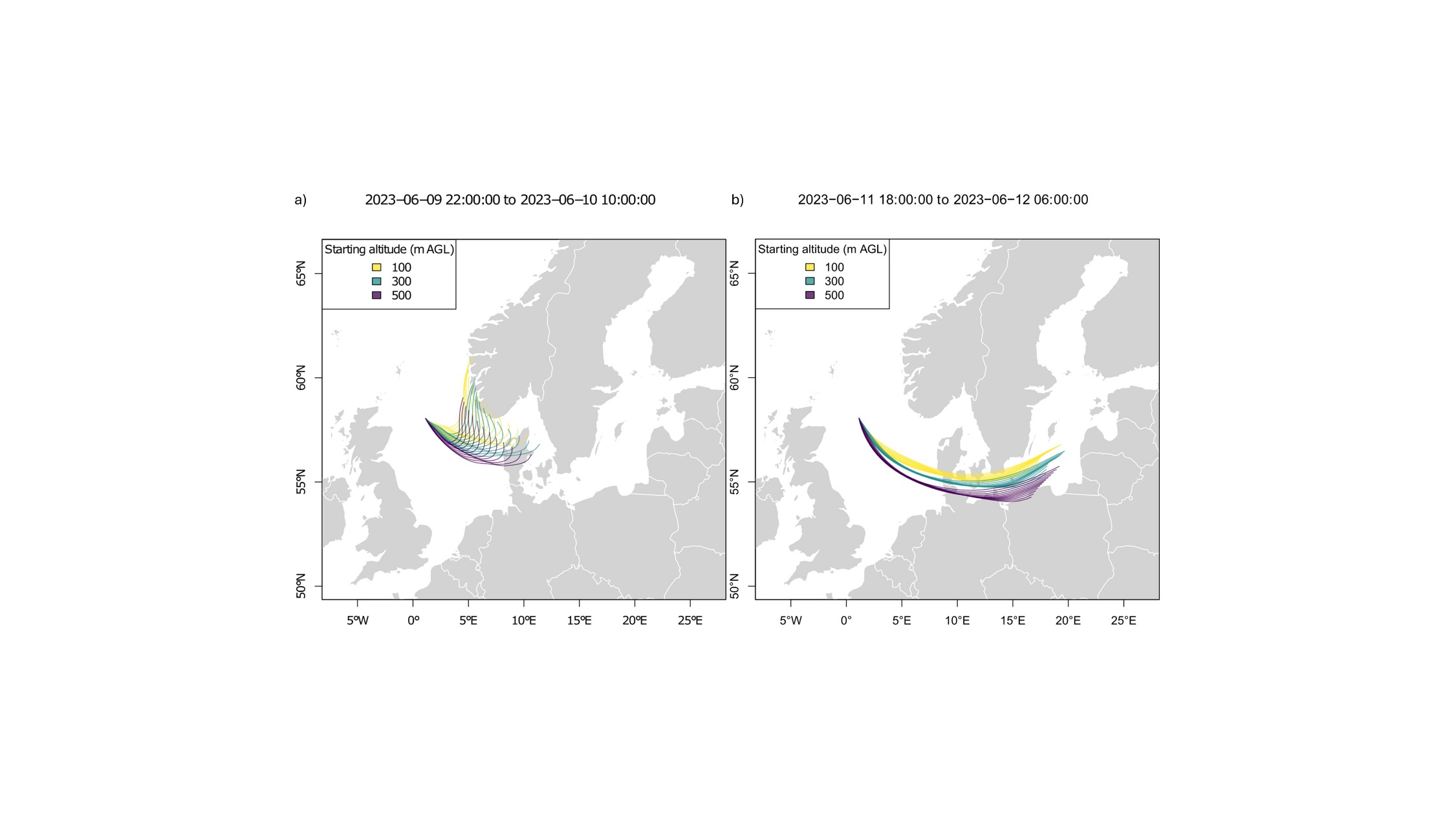


Figure S3. Backward wind trajectories-based arrival times of hoverfly movement events onto the oil rig for the (a) 10^th of^ June 2023 and (b) 12^th^ of June 2023 movement events. Run-time of 36 hours, with one trajectory calculated for every hour, 12 hrs before arrival time at the oil rig. Colours show altitude (yellow, 100; green, 300; purple, 500 m AGL)
